# Healing Outcomes in Diabetic Foot Ulcers Managed Within a Structured Multidisciplinary Care Model: A Retrospective Study

**DOI:** 10.1155/jdr/1730605

**Published:** 2026-07-24

**Authors:** Gadadharan Vijayakumar, Jazeela Abdul Rahim, Gopika Satheesh, G. K. Sreehari, V. Aswathi, K. Sreekanth, V. Revathi, C. S. Vishnu Narayanan, Aswathy Gopan, Abdul Jaleel

**Affiliations:** ^1^ Medical Trust Hospital and Diabetes Care Centre, Pathanamthitta, Kerala, India; ^2^ Cardiovascular Diseases and Diabetes Biology, BRIC-Rajiv Gandhi Centre for Biotechnology (BRIC-RGCB), Thiruvananthapuram, Kerala, India; ^3^ Manipal Academy of Higher Education, Manipal, Karnataka, India, manipal.edu

**Keywords:** comorbidity, debridement, diabetes mellitus, podiatry, revascularization, treatment outcome

## Abstract

**Background:**

Diabetic foot ulcers (DFU) impose a healthcare burden due to prolonged hospitalization, high amputation rates, and increased mortality. This study evaluated healing outcomes and explored factors associated with adverse outcomes among patients managed within a structured multidisciplinary DFU care setting at a specialized diabetes care center in Kerala.

**Methods:**

A retrospective analysis was conducted among 1057 patients with DFUs managed between January and November 2022. Care included wound culture‐directed antibiotics, glycemic optimization, offloading, daily debridement and dressing, and vascular assessment when indicated. Healing outcomes were assessed, and factors associated with poor outcomes and delayed healing were evaluated using multivariable logistic regression.

**Results:**

In the full cohort, 74.7% of patients achieved complete healing, whereas the major amputation rate was 2.6%. After excluding referrals, deaths, and loss to follow‐up, 887 patients comprised the complete follow‐up cohort, of whom 790 (89.1%) achieved favorable outcomes, and 97 (10.9%) experienced poor outcomes (nonhealing ulcer or major amputation). Coronary artery disease (OR 2.08, 95% CI 1.23–3.51; *p* = 0.006) and prior ulcer history (OR 2.41, 95% CI 1.55–3.74; *p* < 0.001) were independently associated with poor outcomes. Among patients with healing‐time data (*n* = 766), chronic kidney disease was independently associated with delayed healing (> 5 months) (OR 1.96, 95% CI 1.26–3.06; *p* = 0.003).

**Conclusion:**

High rates of favorable outcomes and low rates of major amputation were observed in this cohort managed within a multidisciplinary DFU care model. These findings support the need for prospective multicenter evaluations of multidisciplinary DFU care models.

## 1. Background

Diabetic foot is a long‐term complication of diabetes, characterized by deep tissue lesions often linked to neuropathy and peripheral vascular disease in the lower extremities [1]. A common manifestation of this condition is diabetic foot ulcers (DFUs), with a lifetime incidence of 15%–25% among individuals with diabetes [2]. The global prevalence of DFU among individuals with diabetes is estimated at 6.3%, with a prevalence rate of 5.5% in Asia. India ranks fifth worldwide, with a prevalence of 11.6% [3]. DFUs pose a significant economic burden, accounting for approximately 50% of all diabetes‐related hospitalizations [4–6]. A recent Indian cohort study reported that higher ulcer grade, larger ulcer area, longer duration, and a history of previous ulcers were associated with greater treatment costs. Moreover, care in private facilities or from skilled practitioners further increased expenses, and nearly two‐thirds of DFU‐related hospital admissions resulted in catastrophic health expenditure [7]. Given these expenditures and the increasing global prevalence of diabetes, DFUs represent a critical public health concern [2].

DFUs can result in severe complications, including infections, prolonged hospitalizations, amputations of lower extremities, and increased mortality. Major amputations represent one of the most severe adverse outcomes of DFUs and are frequently evaluated alongside nonhealing when assessing prognosis and treatment outcomes. Neuropathy may prevent patients from recognizing the onset of their ulcers, leading to delayed intervention and worsening outcomes such as severe infections, gangrene, and amputations [8]. Several factors contribute to delayed healing, including diabetic neuropathy, peripheral vascular disease, foot deformities, male sex, Type 2 diabetes mellitus, older age, chronic hyperglycemia, previous history of foot ulceration, prolonged duration of Type 2 diabetes mellitus, and low health literacy [2]. Additionally, comorbidities such as cardiovascular disease and nephropathy further delay healing [8]. A recent meta‐analysis identified low ankle‐brachial index (ABI), low physical activity, smoking, depression, and other factors as significant predictors of poor DFU outcomes [9]. Beyond physiological factors, psychological stress, anxiety, and depression may also influence wound healing and DFU prognosis [2].

Ulcer recurrence remains a serious concern, with more than 40% of patients experiencing reulceration. Following an initial amputation, the risk of subsequent amputations may increase up to threefold [10]. Moreover, diabetic foot infections are among the most challenging complications, requiring coordinated multidisciplinary management to reduce recurrence and improve outcomes [11].

Effective DFU management requires a comprehensive approach that considers diabetes‐related complications, ulcer characteristics, and patient adherence to treatment [2]. Key strategies such as pressure offloading, meticulous debridement, effective infection management, and timely revascularization are essential components of care [12]. Optimal glycemic control through pharmacological interventions, dietary control, and physical activity is also critical, alongside routine podiatric evaluations and comprehensive management of associated comorbidities [4, 8, 13]. The 2024 Core Outcome Set for DFU trials standardized definitions for key outcomes, improving consistency across studies [14]. Early detection, prompt treatment, and consistent follow‐up remain crucial for mitigating the global burden of DFUs and minimizing amputation rates [15]. Delayed healing is also clinically important, as prolonged ulcer duration increases the risk of infection, hospitalization, recurrent ulceration, and amputation.

A recent regional review highlights persistent challenges in diabetic foot care across South‐East Asia and India, emphasizing the need for enhanced awareness, specialized foot clinics, affordable wound‐care and offloading interventions, and regional collaboration to curb the high burden of DFUs [16]. In Kerala, due to the high incidence of diabetes [17], research focused on DFUs is particularly relevant. Although multiple treatment modalities exist for the management of DFUs, variations in patient outcomes have been reported with different therapeutic approaches [18]. A recent review noted that improving DFU management guidelines remains challenging due to a scarcity of high‐quality research evidence [11]. Hospital registry studies offer valuable insights into the clinical profiles and outcomes of patients with DFUs, helping to refine interventions and evaluate outcomes under guideline‐based care. However, most studies emphasize risk factors and complications, with limited evaluation of healing outcomes under standardized treatment protocols.

Although India bears a substantial burden of DFUs, many studies are constrained by small sample sizes, limiting generalizability. Although multicentric studies are ideal, well‐designed single‐center research with adequate sample sizes and structured multidisciplinary care protocols can also provide valuable clinical evidence. To address this gap, the present study, conducted in a specialized diabetes care hospital, evaluates DFU treatment outcomes under structured multidisciplinary care and, as a secondary analysis, explores factors associated with poor outcomes (nonhealing or major amputation) and delayed healing. The findings are aimed at generating clinically relevant insights that may inform improved management strategies in the Indian context.

## 2. Materials and Methods

### 2.1. Study Design and Population

This retrospective study utilized the inpatient registry of a specialized diabetic care center in Kerala. A total of 1057 patients admitted with DFUs between January and November 2022 were included. Ethical approval was obtained from the Medical Trust Hospital and Diabetes Care Center, Kerala.

For patients with multiple admissions, only instances involving new ulcers after complete healing of the previous ones were considered, whereas follow‐up admissions were excluded. In patients with multiple ulcers, the most severe ulcer was documented. Outpatient cases were excluded from the analysis. As this was a retrospective study, no a priori sample size calculation was performed; the study size was determined by available records [19].

### 2.2. Structured Multidisciplinary Care

#### 2.2.1. Assessment and Admission Protocol

Initial assessments included evaluation of wound status (size, depth, presence of exudate, and signs of infection) and peripheral circulation using the ABI. Comorbidities such as hypertension, dyslipidemia (DLP), cardiovascular, renal, endocrine, or hormonal abnormalities, and complications including critical limb ischemia, osteomyelitis, and sepsis were systematically documented.

Routine investigations included a complete blood count, erythrocyte sedimentation rate (ESR), C‐reactive protein (CRP), serum creatinine for renal function, a lipid profile, HbA1c, and x‐rays of the affected part to detect osteomyelitis or foreign bodies. Additional tests, including pus culture and sensitivity, liver function, and serum electrolytes, were performed as required. Broad‐spectrum intravenous antibiotics were initiated empirically and adjusted based on sensitivity reports. The indications for culture testing were based on clinical suspicion of infection, including purulent discharge or signs of systemic or local infection. Patients with critical ischemia or extending gangrene underwent peripheral revascularization after ensuring adequate renal function and infection control. Strict offloading measures were implemented to minimize pressure on the affected area, and patients and caregivers received education on foot care.

#### 2.2.2. Medical Management and Wound Care

Anti‐hypertensive medications, statins, and other necessary drugs were continued, whereas patients with peripheral vascular disease received dual antiplatelet therapy, cilostazol, and antithrombotics. Vital parameters such as temperature, blood pressure, renal function, white blood cell count, and hemoglobin levels were regularly monitored to guide treatment modifications.

At the time of admission, most patients presented with markedly elevated HbA1c levels, typically exceeding 9%–10%. Most patients required initiation of insulin during hospitalization due to poor baseline glycemic control. Blood glucose was monitored at least three times daily, and the hospital nutritionist provided a strict diabetic diet. Glycemic control was managed with oral hypoglycemic agents or insulin, with individualized dose adjustments based on frequent monitoring. Patients were advised to continue insulin postdischarge.

Diabetic educators provided structured training on self‐monitoring of blood glucose (SMBG) and insulin titration, and patients were instructed to maintain daily records. During follow‐up reviews, glycemic control over the preceding week was closely assessed, and therapy was adjusted as needed. Since most ulcers healed within approximately 4 months, repeat HbA1c testing was required only in a limited number of cases. Therefore, HbA1c values were primarily considered as baseline measures in subsequent analyses. Fasting blood sugar (FBS) and random blood sugar (RBS) values recorded during follow‐up were used to assess short‐term glycemic control achieved under the multidisciplinary care protocol.

Empirical broad‐spectrum intravenous antibiotics were initiated and subsequently modified according to culture sensitivity reports. Wound care protocol included simple dressings with metronidazole gel, papain urea ointment, or cadexomer iodine cream, depending on the ulcer type.

Daily wound debridement and dressing were performed using sterile techniques. Wounds were cleaned with normal saline and dressed with appropriate antibiotic ointments or advanced wound‐care materials, then covered with gauze and bandages. Vacuum‐assisted therapy was employed when necessary. Patients were usually hospitalized for 7–21 days, depending on the severity of the infection.

At discharge, patients were prescribed culture‐sensitive oral antibiotics in addition to other medications. Emphasis was placed on individualized offloading measures, including total contact casts, posterior slabs, pneumatic walkers, or adapted footwear. Caregivers were educated on proper wound dressing techniques and dressing‐change frequency based on wound type and dressing modality. Dressing materials were provided to cover the period until the next scheduled review. Additionally, both patients and caregivers received education on proper insulin adjustment.

#### 2.2.3. Follow‐Up and Long‐Term Care

Wound healing was initially monitored weekly, with the frequency of assessments reduced as healing progressed. During follow‐up visits, ulcer characteristics, including size, depth, and signs of healing, were reassessed. Adjustments to antibiotics, dressing regimens, and glycemic management were made as required. Adherence to offloading measures was assessed and reinforced. Upon complete healing, patients received customized footwear and reinforced foot‐care education. Long‐term follow‐up emphasized podiatric care and strict maintenance of glycemic control.

#### 2.2.4. Data Recording

We collected sociodemographic and diabetes‐related information, including gender, age, occupation, smoking and alcohol use, duration of diabetes, HbA1c levels, method of antidiabetic treatment, and presence of comorbidities such as hypertension, DLP, coronary artery disease (CAD), chronic kidney disease (CKD), and thyroid abnormalities. Previous history of ulcers, amputations, foot care awareness, and practices were also assessed.

Data on the development and progression of ulcers, including site, duration, treatment methods, healing outcomes, and follow‐up outcomes, were documented through case sheets and patient follow‐up calls. Outcomes such as ulcer healing, lower extremity amputation (LEA) levels, and healing time were examined. LEA was categorized as above‐knee amputation and below‐knee amputation. Data collection concluded in December 2023.

The primary outcome was a poor outcome, defined as nonhealing or major amputation among patients with complete follow‐up. A favorable outcome was defined as healing without major amputation. Nonhealing was defined as failure to achieve complete epithelialization during the follow‐up period. Major amputation was classified as a poor outcome irrespective of stump healing. Delayed healing was defined as ulcer healing duration exceeding 5 months and was assessed only among patients with available healing‐time data. Factors associated with poor outcomes and delayed healing were examined as secondary analyses.

##### 2.3. Statistical Analysis

Data entry was performed using Epidata 3.12 [20], and statistical analyses were performed using IBM SPSS Statistics for Windows, Version 21.0 (IBM Corp., Armonk, New York, 2012). Normality of continuous variables was assessed using the Kolmogorov–Smirnov test. Continuous variables were summarized as mean ± SD or median, as appropriate, whereas categorical variables were expressed as frequencies and percentages. Analyses were conducted using available complete data for each model, and the number of participants included in each analysis was reported.

To assess potential selection bias, baseline characteristics were compared between patients included in the complete follow‐up cohort and those excluded due to referral, death, or loss to follow‐up. Additionally, a worst‐case sensitivity analysis was performed in the full cohort by classifying deaths, referrals, loss to follow‐up, major amputations, and nonhealing ulcers as poor outcomes.

Associations between patient characteristics and healing outcomes were evaluated using univariate analyses. The Pearson chi‐squared test was applied for categorical variables, whereas the Mann–Whitney *U* test was used for continuous variables.

Multivariable binary logistic regression was performed to identify factors independently associated with poor outcome and delayed healing, with results expressed as odds ratios (ORs) and 95% confidence intervals (CIs). Variables were selected a priori based on clinical relevance and prior evidence, and were entered simultaneously into the multivariable models.

For the primary poor‐outcome model, the analysis was conducted on the complete follow‐up cohort (*n* = 887). HbA1c was excluded from the primary multivariable model because of substantial missing data and was evaluated separately in sensitivity analyses using both categorical and continuous HbA1c variables. To evaluate changes in glycemic control during follow‐up, baseline and final‐review FBS and RBS values were compared among patients with available paired measurements using paired *t*‐tests. Since repeat HbA1c measurements were available only in a limited number of patients, longitudinal changes in HbA1c were not assessed.

For delayed healing analysis, only patients with available healing time data (*n* = 766) were included. Antibiotic use was excluded from the primary delayed‐healing multivariable model because of potential confounding by indication and counterintuitive directionality and was evaluated separately in sensitivity analysis.

Clinically overlapping variables were evaluated during model building to minimize redundancy. Model adequacy was assessed using the events‐per‐variable (EPV) criterion, with separate EPV values calculated for each model based on the number of variables entered. An EPV ≥ 10 was considered acceptable for model stability and bias reduction [21, 22]. Model performance was further assessed using receiver operating characteristic curve analysis with area under the curve (AUC) and the Hosmer–Lemeshow goodness‐of‐fit test.

Additional chi‐squared analyses were performed to evaluate associations between healing outcomes and variables including HbA1c category (< 8% vs. ≥ 8%), duration of diabetes, occupation, smoking and alcohol use, footwear type, offloading technique, and type of antidiabetic regimen. Diabetes‐related complication burden was quantified using a four‐level composite score (0, 1, 2–3, and 4–5 complications) based on neuropathy, nephropathy, peripheral artery disease (PAD), retinopathy, and DLP, and evaluated using chi‐squared and unadjusted logistic regression analyses. A *p* value < 0.05 was considered significant.

## 3. Results

### 3.1. Patient Profile

In the study population, over 60% were aged 50–69 years, with males comprising 70%. The average duration of diabetes was 16.45 ± 9.23 years, and nearly 65% had diabetes for more than a decade. A total of 79.4% of patients had a family history of diabetes mellitus. The average HbA1c level was 10.0*%* ± 2.17*%*, indicating poor baseline glycemic control in most patients. Most patients were receiving insulin and oral antidiabetic medications.

Among comorbidities, 57.9% of patients had hypertension, 43% had DLP, 24.7% had CAD, 16.4% had CKD, 7.4% had thyroid abnormalities, and 12.6% had retinopathy (Table 1).

**Table 1:** Demographic details of the study participants.

Variables	Categories	Number (%)
Age group	≤ 39 years	18 (1.7%)
	40–49 years	116 (11%)
	50–59 years	267 (25.3%)
	60–69 years	376 (35.6%)
	70–79 years	217 (20.5%)
	≥ 80 years	63 (6%)

Sex	Male	740 (70%)
	Female	317 (30%)

Duration of Type 2 diabetes mellitus	≤ 5 years	118 (11.2%)
	6–10 years	216 (20.4%)
	11–15 years	214 (20.3%)
	16–20 years	237 (22.4%)
	≥ 21 years	272 (25.7%)

Type 2 diabetes mellitus onset	≤ 30 years	85 (8%)
	31–40 years	281 (26.6%)
	41–50 years	333 (31.5%)
	51–60 years	245 (23.2%)
	> 60 years	113 (10.7%)

Comorbidities	HTN	612 (57.9%)
	DLP	454 (43%)
	CAD	261 (24.7%)
	CKD	173 (16.4%)
	Thyroid abnormalities	78 (7.4%)
	Retinopathy	133 (12.6%)

Family history of Type 2 diabetes mellitus	Yes	839 (79.4%)
	No	218 (20.6%)

### 3.2. Ulcer Profile

Approximately 21% of ulcers were attributed to injury or trauma, 13.6% to blisters, 8.1% to calluses, 10.5% to abscesses, whereas 34.2% were of unknown origin. The median duration of ulcer was 30 days, with 70% presenting ulcers within 2 months. 42% of the patients had a history of foot ulcers, 9.6% had a history of amputation, and 5% had bilateral ulcers at the time of admission. Only 15% of patients reported using specialized footwear.

Ulcer cultures were performed in 46% of the patients, with microbial growth detected in 99.5% of those cases. Gram‐negative strains were more prevalent (64.8%) compared with gram‐positive isolates. The predominant microorganisms identified were *Enterococcus faecalis* (gram‐positive) and *Pseudomonas aeruginosa* (gram‐negative) (Table S1). The most common sites of ulcers were the toes and sole, followed by other regions of the foot (Table S2).

### 3.3. Types of DFUs

Neuropathic ulcers were the most common (76%), followed by neuro‐ischemic (18.5%) and ischemic (5.4%). Moderate‐to‐severe neuropathy was identified in 77.3% of patients. Overall, 18.7% underwent amputation, including 15.4% minor toe amputations and 2.7% major amputations.

ABI assessment showed that 46.9% had ABI values between 0.9 and 1.4, whereas 21.8% had ABI values between 0.6 and 0.9, and 11.2% had limb‐threatening ischemia (ABI < 0.6) (Table S3).

### 3.4. Ulcer Healing Outcomes

At admission, 72% (*n* = 761) of patients were already receiving antibiotics. The average healing time for ulcers was 4.6 ± 3.1 months, with a median of 4 months. Patients had an average hospital stay of 11.75 ± 5.3 days and attended 6.48 ± 5.22 follow‐up visits postdischarge. Approximately 10% required readmission due to recurrence of the index ulcer or development of new ulcers.

**Figure 1:**
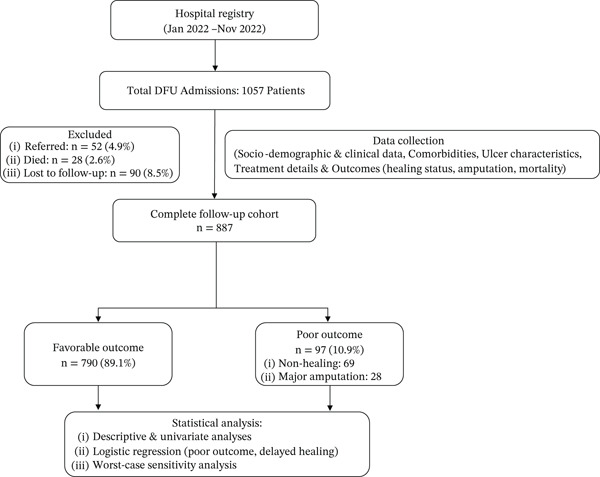
Study flow diagram showing participant inclusion, follow‐up outcomes, and analytical cohorts. Primary analysis was performed in the complete follow‐up cohort (*n* = 887), in which poor outcome was defined as nonhealing or major amputation. Delayed healing (> 5 months) was evaluated among participants with available healing‐time data (*n* = 766). A worst‐case sensitivity analysis was conducted in the full cohort (*n* = 1057).

Among the 1057 patients, 74.7% achieved complete healing, whereas 6.5% had nonhealing ulcers. Major amputation and mortality rates were both 2.6%, whereas 4.9% were referred and 8.5% were lost to follow‐up (Table S4). Figure 1 illustrates participant flow, follow‐up, and outcomes. Figure S1 presents photographic documentation of ulcer healing in selected cases.

### 3.5. Glycemic Control During Follow‐Up

Among patients in the complete follow‐up cohort with available paired measurements, glycemic parameters improved significantly from admission to final review. FBS decreased from 188.19 ± 84.92 to 141.32 ± 39.30 mg/dL, with a mean reduction of 46.87 mg/dL (*p* < 0.001). RBS decreased from 269.37 ± 114.41 to 216.57 ± 82.23 mg/dL, with a mean reduction of 52.81 mg/dL (*p* < 0.001) (Table S5). These findings show significant reductions in short‐term glycemic parameters during follow‐up in this care setting.

### 3.6. Sample Size, Cohort Characteristics, and Sensitivity Analysis

Of the 1057 patients initially enrolled, 170 were excluded from the complete follow‐up cohort because of referral (*n* = 52), death (*n* = 28), or loss to follow‐up (*n* = 90), leaving 887 participants for the primary outcome analysis (Figure 1).

Within the complete follow‐up cohort, 790 (89.1%) achieved favorable outcomes, whereas 97 (10.9%) had poor outcomes, defined as nonhealing or major amputation.

Baseline characteristics were compared between included (*n* = 887) and excluded (*n* = 170) participants to assess potential selection bias. Excluded patients were significantly older and had a higher prevalence of CAD and CKD, and differed in sex distribution. No significant differences were observed for DLP, HbA1c levels, or prior ulcer history (Table S6).

A worst‐case sensitivity analysis, performed in the full cohort by classifying deaths, referrals, loss to follow‐up, major amputations, and nonhealing ulcers as poor outcomes, yielded a favorable outcome rate of 74.7%, compared with 89.1% in the complete follow‐up cohort, suggesting potential overestimation of favorable outcomes after exclusion of higher‐risk patients.

EPV assessment demonstrated adequate model stability for the primary poor outcome, delayed healing, and worst‐case sensitivity models. The HbA1c sensitivity model demonstrated borderline EPV due to a reduced sample size resulting from missing HbA1c data (Table S7).

### 3.7. Factors Associated With Healing Outcomes and Healing Time

Associations between clinical variables and healing outcomes were initially explored using chi‐squared and nonparametric analyses, followed by multivariable logistic regression. In the primary multivariable logistic regression model, CAD (OR 2.08, 95% CI 1.23–3.51, *p* = 0.006) and past ulcer history (OR 2.41, 95% CI 1.55–3.74, *p* < 0.001) were independently associated with poor outcome. Age, sex, hypertension, CKD, and DLP were not significantly associated with poor outcome (Table 2). Model performance demonstrated modest discrimination (AUC = 0.656; 95% CI: 0.599–0.713) with acceptable calibration (Hosmer–Lemeshow *p* = 0.895).

**Table 2:** Multivariable logistic regression analysis of factors associated with poor outcome in diabetic foot ulcers.

Variable	Categories (reference)	Adjusted OR (95% CI)	*p*
Age (years)	Continuous	0.99 (0.97–1.01)	0.380
Sex	Male versus female	1.19 (0.72–1.96)	0.496
Hypertension	Yes versus no	1.25 (0.74–2.12)	0.404
Coronary artery disease (CAD)	Yes versus no	2.08 (1.23–3.51)	0.006
Chronic kidney disease (CKD)	Yes versus no	0.86 (0.46–1.59)	0.619
Dyslipidemia (DLP)	Yes versus no	0.79 (0.47–1.31)	0.354
Past ulcer history	Yes versus no	2.41 (1.55–3.74)	< 0.001

*Note:*Outcome variable: poor outcome (nonhealing ulcer or major amputation) versus favorable outcome (healed ulcer) among participants with complete follow‐up (*n* = 887). ORs > 1 indicate higher odds of a poor outcome. Variables were entered simultaneously using the enter method. Model performance:*A* *U* *C* = 0.656(95% CI: 0.599–0.713); Hosmer–Lemeshow*p* = 0.895.

HbA1c was evaluated separately in sensitivity analyses because of substantial missing data. In this sensitivity analysis, HbA1c ≥ 8% was not significantly associated with poor outcomes.

Mann–Whitney *U* test revealed significant differences in ulcer duration (*p* value = 0.001), hospital stay length (*p* value < 0.001), the number of follow‐up reviews (*p* value = 0.001), and HbA1c levels (*p* value = 0.039) between favorable and poor outcome groups (Table S8).

Additional chi‐square analyses demonstrated significant associations between healing outcomes and ABI category (*χ* ^2^ = 8.82, *p* = 0.032), footwear type (*χ* ^2^ = 14.22, *p* = 0.014), and offloading type (*χ* ^2^ = 10.21, *p* = 0.017). Smoking, alcohol use, occupation, duration of diabetes, antidiabetic treatment regimen, and HbA1c category (< 8% versus ≥ 8%) were not significantly associated with healing outcomes (Table S9).

Complication burden (0, 1, 2–3, and 4–5 complications) did not demonstrate a significant association with healing outcomes (*χ* ^2^ = 1.57, *p* = 0.666) (Table S10). Similarly, unadjusted logistic regression analysis showed no significant association between complication burden and poor outcome (Table S11).

#### 3.8. Healing Time Analysis and Delayed Healing

Delayed healing, defined as a healing duration exceeding 5 months, was assessed among patients with available healing time data (*n* = 766). Univariate analysis showed that delayed healing was significantly associated with antibiotic usage, CKD, peripheral neuropathy, nephropathy, PAD, CAD, retinopathy, DLP, and type of ulcer (Table S12).

In multivariable logistic regression analysis, CKD was independently associated with delayed healing (OR 1.96, 95% CI: 1.26–3.06, *p* = 0.003), whereas retinopathy was associated with lower odds of delayed healing (OR 0.46, 95% CI 0.27–0.77, *p* = 0.003). PAD demonstrated a borderline association with delayed healing (OR 4.93, 95% CI 0.92–26.58, *p* = 0.063). Other variables, including age, sex, hypertension, CAD, DLP, peripheral neuropathy, and ulcer type, were not independently associated with delayed healing (Table 3).

**Table 3:** Multivariable logistic regression analysis of factors associated with delayed healing in diabetic foot ulcers.

Variable	Categories (reference)	Adjusted OR (95% CI)	*p*
Age	Continuous	1.01 (1.00–1.03)	0.072
Sex	Male versus female	0.98 (0.69–1.39)	0.895
Past ulcer history	Yes versus no	1.14 (0.83–1.58)	0.416
Hypertension	Yes versus no	0.80 (0.55–1.16)	0.243
Coronary artery disease (CAD)	Yes versus no	1.21 (0.80–1.83)	0.377
Chronic kidney disease (CKD)	Yes versus no	1.96 (1.26–3.06)	0.003
Dyslipidemia (DLP)	Yes versus no	1.23 (0.85–1.76)	0.275
Retinopathy	Yes versus no	0.46 (0.27–0.77)	0.003
Peripheral neuropathy	Overall variable	—	0.507
Moderate	Versus no/mild	2.31 (0.52–10.34)	0.273
Severe	Versus no/mild	2.27 (0.57–9.01)	0.245
Peripheral artery disease (PAD)	Yes versus no	4.93 (0.92–26.58)	0.063
Type of ulcer	Overall variable	—	0.465
Ischemic ulcer	Versus neuropathic ulcer	0.40 (0.06–2.61)	0.336
Neuro‐ischemic ulcer	Versus neuropathic ulcer	0.25 (0.05–1.38)	0.111
Others	Versus neuropathic ulcer	1.33 (0.33–5.43)	0.689

*Note:*Outcome variable: delayed healing, defined as healing time > 5 months, among patients with available healing‐time data (*n* = 766). ORs > 1 indicate higher odds of delayed healing. Variables were entered simultaneously using the enter method. Antibiotic use was excluded from the primary delayed‐healing model because of potential confounding by indication and counterintuitive directionality; it was evaluated separately in sensitivity analysis. Model performance:*A* *U* *C* = 0.639(95% CI: 0.598–0.679); Hosmer–Lemeshow*p* = 0.228.

Mann–Whitney *U* test showed that patients with delayed healing had significantly longer ulcer duration, longer hospital stay, more follow‐up visits, and longer overall healing times (all *p* < 0.001) (Table S13).

## 4. Discussion

This study represents a large single‐center investigation of DFU outcomes in India and provides insights into healing patterns within a structured multidisciplinary care setting. The overall healing rate was 74.7% in the full cohort and 89.1% in the complete follow‐up cohort after excluding deaths, referrals, and loss to follow‐up. Sensitivity analyses indicated that excluding patients with incomplete follow‐up affected the outcome estimates, underscoring the importance of reporting both cohort‐level and complete follow‐up results.

The observed healing rates were higher than those reported in many previous cohorts, in which healing rates were below 30% within 20 weeks under standard care [12], and exceeded findings from several studies with 1‐year follow‐up [23, 24]. These results align with only a limited number of studies reporting similarly favorable outcomes [25]. Differences across studies likely reflect variation in patient populations, ulcer severity, treatment intensity, follow‐up completeness, and healthcare settings, including adherence to multidisciplinary, evidence‐based DFU management approaches recommended by the International Working Group on the Diabetic Foot (IWGDF) and Infectious Diseases Society of America (IDSA) guidelines [26].

Core components of DFU management, such as glycemic control, pressure offloading, meticulous debridement, infection management, and timely revascularization, are central to evidence‐based DFU care [12]. In the present cohort, the offloading technique was associated with healing outcomes in univariate analysis, highlighting the importance of pressure redistribution strategies. Vacuum‐assisted closure (VAC) therapy was also used in selected cases as part of routine wound management.

Most patients were already receiving antibiotics before admission. In sensitivity analyses, antibiotic use was associated with lower odds of delayed healing, although this finding may reflect differences in infection severity and treatment patterns. Timely identification of PAD and early revascularization remain important components of DFU care, as impaired perfusion can adversely affect wound healing and treatment response [27].

Daily debridement and structured wound care formed part of the treatment protocol. Although current evidence supports weekly debridement [28], more frequent debridement may facilitate a clean wound bed and limit biofilm reformation, which can occur within 24–48 hours. However, the optimal frequency of debridement remains uncertain and warrants further investigation. Structured education for patients and caregivers may have supported treatment adherence and long‐term foot care. Our findings are consistent with prior studies demonstrating that multidisciplinary care and patient education are associated with reduced healing times, amputation rates, and amputation severity [29].

CAD was independently associated with poor outcome, consistent with its established role in impairing tissue perfusion [8, 30–32]. Patients with a previous history of foot ulcer were also more likely to experience poor outcomes, suggesting persistent biomechanical, vascular, or behavioral risk factors that may predispose to recurrent ulceration and impaired healing.

HbA1c was evaluated separately in sensitivity analyses because of substantial missing data. Using an 8% cut‐off, HbA1c was not significantly associated with poor outcome despite extensive evidence linking chronic hyperglycemia to impaired wound healing [5, 8, 33–38]. This may reflect the highly skewed distribution of glycemic status in this cohort, treatment intensification during hospitalization, and the limitations of a single baseline HbA1c measurement in reflecting longitudinal glycemic control. Despite the lack of a significant association between baseline HbA1c and healing outcomes, fasting and random blood glucose levels improved significantly during follow‐up, reflecting short‐term glycemic control with multidisciplinary management.

Delayed healing analysis identified CKD as independently associated with delayed healing, consistent with its known effects on inflammation, endothelial dysfunction, and impaired tissue repair [39–41]. Retinopathy was independently associated with lower odds of delayed healing, although the mechanism underlying this finding remains uncertain and may reflect residual confounding or differences in treatment intensity. Although PAD demonstrated a borderline association with delayed healing, previous studies have consistently linked PAD to impaired healing [23–25, 42]. The wide CI observed in our study may reflect limited precision, whereas the use of ABI may not fully capture microvascular dysfunction relevant to tissue repair. More sensitive measures of local perfusion may provide stronger associations with healing outcomes. Trends toward delayed healing among neuro‐ischemic and ischemic ulcers were consistent with prior studies [32].

Beyond traditional clinical factors, ABI category, footwear type, and offloading technique were associated with healing outcomes in univariate analyses, underscoring the importance of vascular status and adherence to appropriate footwear and pressure‐redistribution strategies.

Although the composite complication burden was not significantly associated with poor outcome, patients with one to three complications showed slightly higher rates of poor outcome than those without complications. The small size of the highest burden group (4–5 complications) may have limited the stability of its estimates. Although not significant, the observed pattern is broadly consistent with the emerging “cardio–renal–metabolic–foot” framework, which conceptualizes DFU as a marker of multisystem deterioration rather than an isolated wound process [43].

However, certain study limitations should be acknowledged. This was a single‐center retrospective study, and the sample size was determined by the availability of clinical records rather than a priori sample calculation. Although the large cohort improves precision and provides valuable clinical data, generalizability may be limited because the study was conducted in a specialized tertiary diabetes care center with intensive inpatient multidisciplinary management.

Attrition due to referral, loss to follow‐up, or death (~16%) may have introduced selection bias and contributed to the overestimation of favorable outcomes, as excluded patients were older and had a higher burden of comorbidities. Although a worst‐case sensitivity analysis was performed, residual bias cannot be excluded. Analyses were conducted using available complete data; substantial missingness in some variables, particularly HbA1c, reduced the sample size for sensitivity analyses and may have affected model stability. Repeat HbA1c measurements were available only for a limited number of patients, precluding comprehensive assessment of long‐term glycemic control.

The relatively small proportion of poor outcomes (10.9%) may have limited statistical power and contributed to unstable estimates for certain variables. Although model performance measures were assessed, formal internal validation procedures, such as bootstrapping or cross‐validation, were not conducted.

Important ulcer severity parameters, including Wagner or WIfI classification, ulcer size, depth, infection severity, and detailed tissue characteristics, were not consistently available and therefore could not be incorporated into the analyses. Residual confounding remains possible because factors such as treatment adherence, health literacy, psychosocial characteristics, nutritional status, and socioeconomic status were not systematically captured. In addition, the structured multidisciplinary care pathway involved multiple concurrent interventions, preventing assessment of the independent contribution of individual treatment components. Potential confounding by indication, particularly regarding antibiotic use, cannot be excluded.

Healing time was reliably recorded only for patients who achieved complete epithelialization; therefore, time‐to‐event and competing‐risk analyses were not feasible. The use of ABI as a measure of vascular status may not fully capture microvascular dysfunction relevant to wound healing. Additionally, the lack of a significant association between HbA1c and poor outcome, together with the observed association between retinopathy and delayed healing, requires further evaluation in prospective studies with longitudinal follow‐up. Finally, the 1‐year follow‐up period did not fully characterize longer‐term outcomes such as ulcer recurrence, late amputations, and mortality.

Future multicenter prospective studies incorporating standardized measures of ulcer severity, longitudinal glycemic assessment, and patient‐centered factors such as adherence, health literacy, and psychosocial determinants are needed to validate and extend these findings.

Our findings highlight the clinical relevance of structured, multidisciplinary DFU care models, in which favorable healing outcomes and low rates of major amputation were observed among patients with DFUs. Factors associated with poor outcomes and delayed healing may help identify patients who could benefit from closer monitoring, targeted interventions, and timely referral to specialized centers.

## 5. Conclusion

This large single‐center analysis of DFU outcomes in India demonstrated high healing rates in both the full cohort and the complete follow‐up cohort, alongside relatively low rates of major amputation. These favorable outcomes were observed among patients managed within a structured, multidisciplinary DFU care setting that incorporated glycemic management, structured offloading, infection management, revascularization, daily debridement, and patient education.

Multivariable analysis identified CAD and prior ulcer history as factors associated with poor outcomes, whereas CKD was associated with delayed healing. These findings highlight the importance of systemic comorbidities and support the concept of DFU as part of a broader cardio–renal–metabolic disease burden rather than an isolated wound condition.

These findings support further evaluation of structured multidisciplinary DFU care models across hospitals and diabetes care centers, particularly in resource‐constrained settings. Factors associated with poor outcomes and delayed healing may inform risk stratification and timely referral to specialist care to optimize DFU management. Future multicenter prospective studies incorporating psychosocial, nutritional, and quality‐of‐life factors will be essential to validate and extend these findings and to further inform evidence‐based DFU care strategies.

**Nomenclature**
ABI: ankle‐brachial index
CAD: coronary artery disease
CKD: chronic kidney disease
CRP: c‐reactive protein
DFU: diabetic foot ulcer
DLP: dyslipidemia
ESR: erythrocyte sedimentation rate
FBS: fasting blood sugar
HbA1c: hemoglobin A1c
LEA: lower extremity amputation
OR: odds ratio
PAD: peripheral artery disease
RBS: random blood sugar
VAC: vacuum‐assisted closure

## Author Contributions

G.V. served as the principal investigator and was responsible for the conception, design, and conduct of the study, as well as the acquisition of clinical data. G.V., J.A.R., and G.S. contributed equally to data analysis, interpretation of findings, and manuscript preparation. G.K.S., V.A., K.S., V.R., C.S.V.N., and A.G. contributed to the conduct of the study and data collection. A.J. was involved in drafting and critically revising the manuscript.

## Funding

No funding was received for this manuscript.

## Disclosure

A.J. had full access to all study data and takes responsibility for the integrity of the data and the accuracy of the analysis. All authors reviewed and approved the final version of the manuscript.

## Ethics Statement

This study was approved by the institutional ethical committee of Medical Trust Hospital and Diabetes Care Centre (IHEC/04/2024/01). All procedures performed in the studies involving human participants were following the institutional and national research committee′s ethical standards and with the 1964 Helsinki declaration and its later amendments or comparable ethical standards. Informed consent was obtained from all the study participants.

## Consent

All patients provided informed consent for the use of clinical photographs.

## Conflicts of Interest

The authors declare no conflicts of interest.

## Supporting Information

Additional supporting information can be found online in the Supporting Information section.

## Supporting information


**Supporting Information 1** STROBE Checklist: Completed STROBE checklist for cohort studies, outlining the reporting compliance of the current manuscript.

**Supporting Information 2** Table S1: Microbiological profile of organisms isolated from diabetic foot ulcers among study participants. Table S2: Distribution of ulcer sites among study participants. Table S3: Ankle‐brachial index (ABI) distribution among study participants. Figure S1: Sequential clinical progression of diabetic foot ulcer (DFU) healing in three patients receiving structured multidisciplinary wound care. Table S4: Distribution of healing outcomes in the full cohort (*n* = 1057). Table S5: Changes in glycemic parameters between admission and final review among patients with complete follow‐up and available paired measurements. Table S6: Comparison of baseline characteristics between included (*n* = 887) and excluded (*n* = 170) participants. Table S7: EPV calculations for logistic regression models. Table S8: Independent‐samples Mann–Whitney *U* test comparing continuous clinical variables between favorable and poor outcome groups. Table S9: Association of clinical, glycemic, vascular, lifestyle, and contextual factors with healing outcomes in diabetic foot ulcers. Table S10: Association between diabetes‐related complication burden and healing outcomes. Table S11: Unadjusted binary logistic regression analysis of complication burden and poor outcome. Table S12: Univariate association of clinical factors with delayed healing (> 5 months). Table S13: Independent‐samples Mann–Whitney *U* test results comparing patients with and without delayed healing.

## Data Availability

The data that support the findings of this study are available from the corresponding author upon reasonable request.
