# Supplementary figures and images for: Healing Outcomes in Diabetic Foot Ulcers Managed Within a Structured Multidisciplinary Care Model: A Retrospective Study
Source: J Diabetes Res. 2026 Jul 24;2026:1730605. doi: 10.1155/jdr/1730605 (PMC13398153; doi:10.1155/jdr/1730605)

## Slide 1
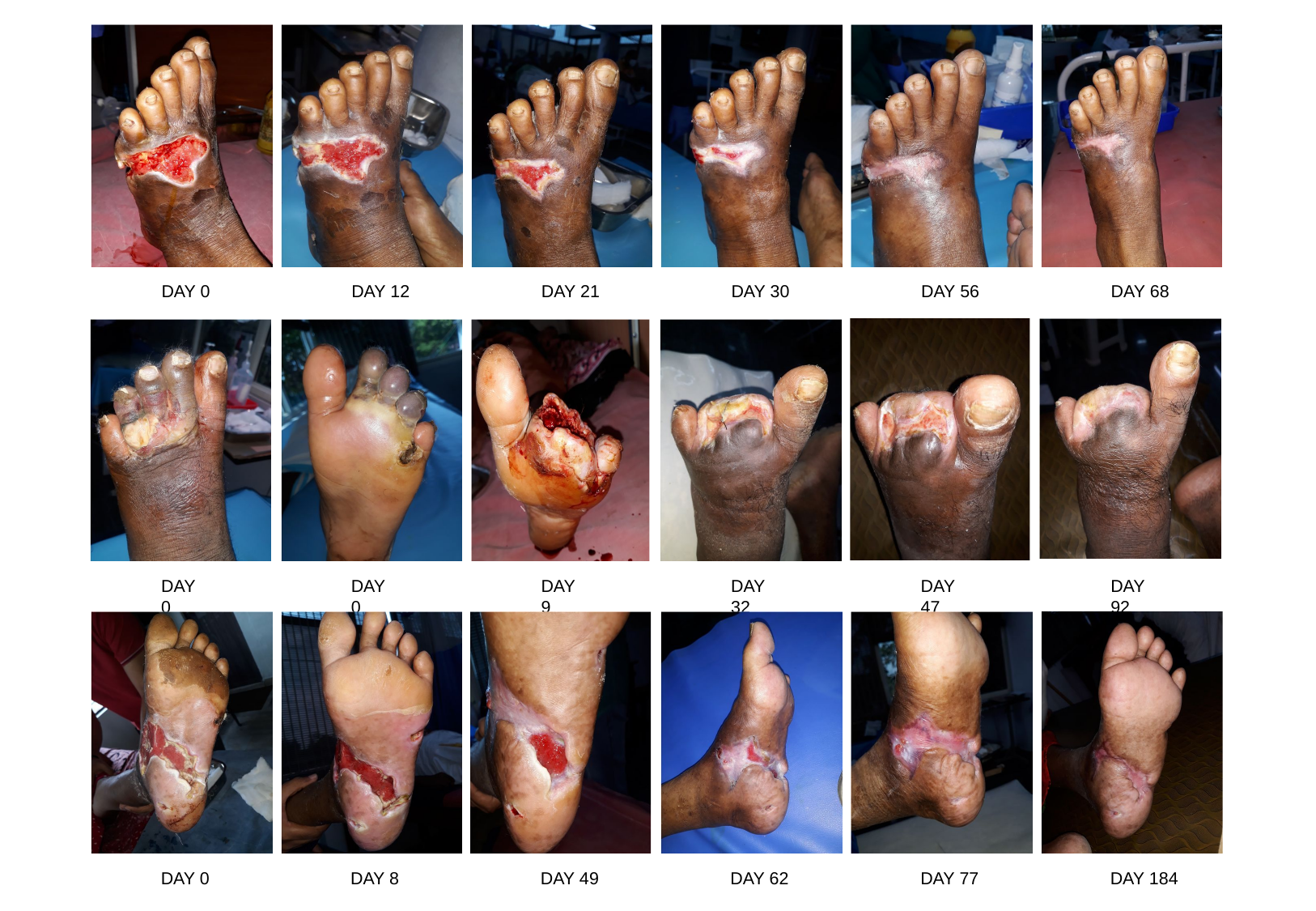

DAY 68
DAY 21
DAY 30
DAY 56
DAY 0
DAY 12
DAY 9
DAY 32
DAY 47
DAY 92
DAY 0
DAY 0
DAY 49
DAY 62
DAY 77
DAY 184
DAY 8
DAY 0

Supplement: Supplementary file 1 — Supporting Information 1 STROBE Checklist: Completed STROBE checklist for cohort studies, outlining the reporting compliance of the current manuscript. [file JDR-2026-1730605-s001.pptx]
