# Supplementary material for: Healing Outcomes in Diabetic Foot Ulcers Managed Within a Structured Multidisciplinary Care Model: A Retrospective Study
Source: J Diabetes Res. 2026 Jul 24;2026:1730605. doi: 10.1155/jdr/1730605 (PMC13398153; doi:10.1155/jdr/1730605)
Supplement: Supplementary file 2 — Supporting Information 2 Table S1: Microbiological profile of organisms isolated from diabetic foot ulcers among study participants. Table S2: Distribution of ulcer sites among study participants. Table S3: Ankle‐brachial index (ABI) distribution among study participants. Figure S1: Sequential clinical progression of diabetic foot ulcer (DFU) healing in three patients receiving structured multidisciplinary wound care. Table S4: Distribution of healing outcomes in the full cohort (n = 1057). Table S5: Changes in glycemic parameters between admission and final review among patients with complete follow‐up and available paired measurements. Table S6: Comparison of baseline characteristics between included (n = 887) and excluded (n = 170) participants. Table S7: EPV calculations for logistic regression models. Table S8: Independent‐samples Mann–Whitney U test comparing continuous clinical variables between favorable and poor outcome groups. Table S9: Association of clinical, glycemic, vascular, lifestyle, and contextual factors with healing outcomes in diabetic foot ulcers. Table S10: Association between diabetes‐related complication burden and healing outcomes. Table S11: Unadjusted binary logistic regression analysis of complication burden and poor outcome. Table S12: Univariate association of clinical factors with delayed healing (> 5 months). Table S13: Independent‐samples Mann–Whitney U test results comparing patients with and without delayed healing. [file JDR-2026-1730605-s002.docx]

**Supplementary Material**

**Healing Outcomes in Diabetic Foot Ulcers Managed within a Structured Multidisciplinary Care Model: A Retrospective Study**

| **Spectrum** | **Number** | **Percentage** |
| --- | --- | --- |
| Gram Negative | 311 | 64.8 |
| Gram positive | 169 | 35.2 |
| **Gram positive** | | |
| *Staphylococcus aureus* | 52 | 10.8 |
| *Staphylococcus haemolyticus* | 17 | 3.5 |
| *Enterococcus faecalis* | 68 | 14.2 |
| *Enterococcus faecium* | 11 | 2.3 |
| **Gram negative** | | |
| *Enterobacter cloacae complex (ECC)* | 38 | 7.9 |
| *Pseudomonas aeruginosa* | 63 | 13.1 |
| *Proteus mirabilis* | 16 | 3.3 |
| *Klebsiella pneumoniae* | 56 | 11.7 |
| *Escherichia coli* | 53 | 11 |
| *Acinetobacter baumannii* | 13 | 2.7 |
| *Citrobacter koseri* | 17 | 3.5 |

**Table S1:** **Microbiological profile of organisms isolated from diabetic foot ulcers among study participants.** Values are presented as frequency and percentage of isolates identified from wound cultures.

| **Site of Ulcer** | **Frequency** | **Percentage** |
| --- | --- | --- |
| Big toes | 236 | 22.4 |
| Other toes | 196 | 18.6 |
| Sole | 144 | 13.6 |
| Heel | 86 | 8.2 |
| Dorsum | 72 | 6.8 |
| Ankle | 70 | 6.6 |
| IDS | 34 | 3.2 |
| Shin | 31 | 2.9 |
| Amputation stump | 65 | 6.3 |
| Others | 121 | 11.4 |

**Table S2:** **Distribution of ulcer sites among study participants.** Values are presented as frequencies and percentages by the anatomical site of ulcer involvement.

| **ABI** | **N (%)** |
| --- | --- |
| > 1.4 | 177 (20.1) |
| 0.9 - 1.4 | 414 (46.9) |
| 0.6 - 0.9 | 192 (21.8) |
| < 0.6 | 99 (11.2) |
| **Total** | **882** |

**Table S3:** **Distribution of ankle–brachial index (ABI) categories among study participants.** ABI categories were classified to reflect the severity of peripheral arterial perfusion status among participants with available ABI measurements.


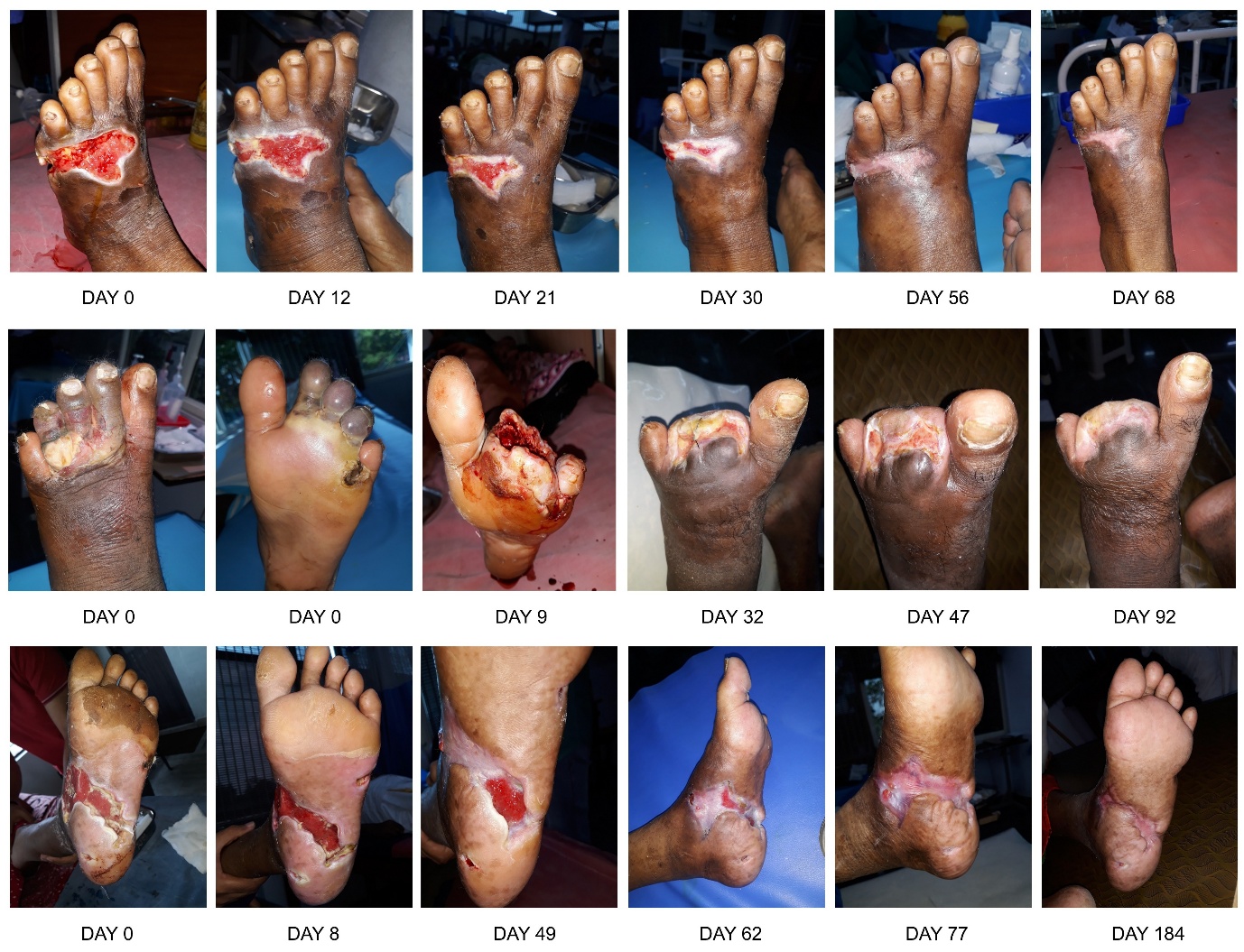


**Fig. S1: Sequential clinical progression of diabetic foot ulcer (DFU) healing in three patients receiving structured multidisciplinary wound care**. Top row (Patient 1): healing of dorsal foot ulcer from Day 0 to Day 68. The images show progressive stages of granulation, epithelialization, and eventual scar formation. Middle row (Patient 2): Healing of a plantar and digital ulcer from Day 0 to Day 92. This patient underwent partial digital amputation, and the images document wound contraction and epithelialization. Bottom row (Patient 3): Healing of a large plantar ulcer located on the heel and midfoot region. Observed from Day 0 to Day 184, the images demonstrate significant tissue regeneration and scar maturation.

| **Outcome** | **n (%)** |
| --- | --- |
| Complete Healing | 790 (74.7%) |
| Non-healed | 69 (6.5%) |
| Death | 28 (2.6%) |
| Referral | 52 (4.9%) |
| Lost to follow-up | 90 (8.5%) |
| Major amputation | 28 (2.6%) |

**Table S4:** **Distribution of healing outcomes in the full cohort (n=1057).**

| **Glycemic parameter** | **Admission, mean ± SD** | **Final review, mean ± SD** | **Mean reduction** | **n** | **p-value** |
| --- | --- | --- | --- | --- | --- |
| FBS (mg/dL) | 188.19 ± 84.92 | 141.32 ± 39.30 | 46.87 | 616 | <0.001 |
| RBS (mg/dL) | 269.37 ± 114.41 | 216.57 ± 82.23 | 52.81 | 129 | <0.001 |

**Table S5**: **Changes in glycemic parameters between admission and final review among patients with complete follow-up and available paired measurements.** Values are presented as mean ± standard deviation (SD). Comparisons between admission and final review values were performed using paired-samples t-tests. Analyses were restricted to patients with complete follow-up and available paired measurements for each parameter.

| **Variable** | **Included (n=887)** | **Excluded (n=170)** | **p-value** |
| --- | --- | --- | --- |
| Age (years, mean ± SD) | 61.8 ± 10.9 | 65.3 ± 11.5 | <0.001 |
| Male (%) | 71.3% | 63.5% | 0.044 |
| CAD (%) | 21.8% | 40.0% | <0.001 |
| CKD (%) | 14.8% | 24.7% | 0.001 |
| DLP (%) | 43.0% | 42.9% | 0.998 |
| HbA1c ≥8% | 83.4% | 77.1% | 0.109 |
| Past ulcer (%) | 41.4% | 45.3% | 0.343 |

**Table S6:** **Comparison of baseline characteristics between included (n=887) and excluded (n=170) participants.** Values are presented as mean ± SD for age and percentages for categorical variables. Excluded participants included those who died, were referred, or were lost to follow-up. FBS, fasting blood sugar; RBS, random blood sugar; SD, standard deviation.

| **Regression model** | **Total sample (n)** | **Events (n)** | **Variables included in model (k)** | **EPV (Events ÷ k)** | **Threshold adequacy** |
| --- | --- | --- | --- | --- | --- |
| Poor outcome model | 887 | 97 | 7 (Age, sex, hypertension, CAD, CKD, DLP, past ulcer history) | 13.9 | Adequate (>10) |
| HbA1c sensitivity model | 626 | 66 | 8 (HbA1c category, age, sex, hypertension, CAD, CKD, DLP, past ulcer history) | 8.3 | Borderline; interpreted cautiously |
| Worst-case poor outcome model | 1057 | 267 | 7 (Age, sex, hypertension, CAD, CKD, DLP, past ulcer history) | 38.1 | Adequate (>10) |
| Delayed healing model | 766 | 260 | 11 (Age, sex, past ulcer history, hypertension, CAD, CKD, DLP, retinopathy, peripheral neuropathy, PAD, type of ulcer) | 23.6 | Adequate (>10) |

**Table S7**: **Events-per-variable (EPV) calculations for logistic regression models****.** EPV was calculated as the number of outcome events divided by the number of variables entered into each multivariable logistic regression model. Poor outcome was defined as non-healing or major amputation. The worst-case sensitivity model additionally classified death, referral, loss to follow-up, major amputations, and non-healing ulcers as poor outcomes. Delayed healing was defined as healing time >5 months among participants with available healing-time data.

| **Variable** | **Total** | **Favorable Outcome Mean Rank** | **Poor Outcome Mean Rank** | **Mann-Whitney U** | **Wilcoxon Rank** | **Asymptotic Sig. (2-sided)** |
| --- | --- | --- | --- | --- | --- | --- |
| **Duration of Ulcer** | 776 | 379.06 | 467.31 | 22218.5 | 262689.5 | 0.001 |
| **Duration of Hospital Stay** | 884 | 428.02 | 561.4 | 26410 | 337276 | <0.001 |
| **Number of Reviews** | 884 | 432.11 | 527.76 | 29639 | 340505 | 0.001 |
| **HbA1c** | 626 | 318.62 | 270.03 | 15611 | 17822 | 0.039 |

**Table S8: Independent-samples Mann–Whitney U test comparing continuous clinical variables between favorable and poor outcome groups.** Poor outcome was defined as non-healing or major amputation. A favorable outcome was defined as healing without major amputation. HbA1c analysis included only participants with available HbA1c data.

| **Variable** | **Categories** | **Favorable Outcome**  **n (%)** | **Poor Outcome**  **n (%)** | **Chi square value (χ²)** | **p-value** |
| --- | --- | --- | --- | --- | --- |
| **HbA1c category** | <8.0% | 89 (85.6%) | 15 (14.4%) | 1.99 | 0.158 |
|  | ≥8.0% | 471 (90.2%) | 51 (9.8%) |  |  |
| **ABI** | <0.6 | 60 (82.2%) | 13 (17.8%) | 8.82 | 0.032 |
|  | 0.6–0.9 | 156 (92.9%) | 12 (7.1%) |  |  |
|  | 0.9–1.4 | 328 (89.4%) | 39 (10.6%) |  |  |
|  | >1.4 | 143 (93.5%) | 10 (6.5%) |  |  |
| **Smoking** | Non-smoker | 531 (90.2%) | 61 (9.8%) | 2.91 | 0.234 |
|  | Ex-smoker | 145 (85.8%) | 24 (14.2%) |  |  |
|  | Smoker | 84 (87.5%) | 12 (12.5%) |  |  |
| **Alcohol** | Non-drinker | 501 (89.1%) | 61 (10.9%) | 0.08 | 0.962 |
|  | Ex-drinker | 154 (88.5%) | 20 (11.5%) |  |  |
|  | Drinker | 135 (89.4%) | 16 (10.6%) |  |  |
| **Occupation** | Not employed | 310 (89.1%) | 38 (10.9%) | 3.73 | 0.588 |
|  | Government/private/self employed | 155 (88.1%) | 21 (11.9%) |  |  |
|  | Farmer/manual labour | 59 (92.2%) | 5 (7.8%) |  |  |
|  | Abroad/business | 55 (91.7%) | 5 (8.3%) |  |  |
|  | Housewife | 136 (90.7%) | 14 (9.3%) |  |  |
|  | Ex-service | 75 (84.3%) | 14 (15.7%) |  |  |
| **Footwear Type** | Plastic | 282 (89.5%) | 33 (10.5%) | 14.22 | 0.014 |
|  | Rubber | 355 (91.3%) | 34 (8.7%) |  |  |
|  | Leather | 40 (90.9%) | 4 (9.1%) |  |  |
|  | Shoes | 5 (100%) | 0 (0%) |  |  |
|  | Special | 104 (80.0%) | 26 (20.0%) |  |  |
|  | No footwear | 4 (100%) | 0 (0%) |  |  |
| **Offloading Type** | No offloading | 271 (84.7%) | 49 (15.3%) | 10.21 | 0.017 |
|  | POP | 5 (62.5%) | 3 (37.5%) |  |  |
|  | Dynacast | 33 (91.7%) | 3 (8.3%) |  |  |
|  | Footwear | 63 (95.5%) | 3 (4.5%) |  |  |
| **Diabetes Duration** | 5 years or below | 97 (93.3%) | 7 (6.7%) | 3.31 | 0.507 |
|  | 6–10 years | 158 (86.8%) | 24 (13.2%) |  |  |
|  | 11–15 years | 164 (88.2%) | 22 (11.8%) |  |  |
|  | 16-20 years | 176 (90.3%) | 19 (9.7%) |  |  |
|  | >20 years | 195 (88.6%) | 25 (11.4%) |  |  |
| **Type of anti-diabetic medication** | OHA | 164 (91.6%) | 15 (8.4%) | 2.94 | 0.402 |
|  | Insulin | 196 (87.9%) | 27 (12.1%) |  |  |
|  | OHA+ Insulin | 413 (89.0%) | 51 (11.0%) |  |  |
|  | diet control / alternate medicines | 17 (81%) | 4 (19%) |  |  |

**Table S9: Association of clinical, glycemic, vascular, lifestyle, and contextual factors with healing outcomes in diabetic foot ulcers.** Values are presented as n (%). Poor outcome was defined as non-healing or major amputation, and a favorable outcome as healing without major amputation, among participants with complete follow-up (n=887). Pearson’s chi-square test was used to compare categorical variables between outcome groups. Percentages represent row percentages within each category. Significant associations were observed for ankle–brachial index (ABI), offloading type, and footwear type.

| **Complication Category** | **Favorable Outcome**  **n (%)** | **Poor Outcome n (%)** | **Chi square value (χ²)** | **p-value** |
| --- | --- | --- | --- | --- |
| 0 – No complication | 88 (89.8%) | 10 (10.2%) | 1.57 | 0.666 |
| 1 – Single complication | 296 (90.0%) | 33 (10.0%) |  |  |
| 2–3 – Moderate complications | 349 (87.7%) | 49 (12.3%) |  |  |
| 4–5 – High complications | 56 (91.8%) | 5 (8.2%) |  |  |

**Table S10: Association between diabetes-related complication burden and healing outcomes.** Poor outcome was defined as non-healing or major amputation. Complication burden was calculated using neuropathy, nephropathy, peripheral artery disease, retinopathy, and dyslipidemia.

| **Variable** | **Categories (Reference)** | **OR (95% CI)** | **p-value** |
| --- | --- | --- | --- |
| Complication category | Overall variable | — | 0.668 |
| No complication | vs High complications (4–5) | 1.27 (0.41–3.92) | 0.674 |
| Single complication | vs High complications (4–5) | 1.25 (0.47–3.34) | 0.658 |
| Moderate complications (2–3) | vs High complications (4–5) | 1.57 (0.60–4.12) | 0.357 |

**Table S11: Unadjusted binary logistic regression analysis of complication burden and poor outcome.** Poor outcome was defined as non-healing or major amputation. High complication burden (4–5 complications) was used as the reference category. Odds ratios >1 indicate higher odds of a poor outcome.

| **Variables** | **Chi square value (χ^2^)** | **Degree of Freedom** | **Asymptotic Sig. (2-sided)** |
| --- | --- | --- | --- |
| Antibiotic Usage | 17.55 | 1 | < 0.01 |
| Retinopathy | 4.48 | 1 | 0.034 |
| Hypertension | 1.16 | 1 | 0.281 |
| Coronary artery disease (CAD) | 4.68 | 1 | 0.031 |
| Chronic kidney disease (CKD) | 11.92 | 1 | 0.001 |
| Dyslipidemia (DLP) | 4.44 | 1 | 0.035 |
| Peripheral Neuropathy | 9.53 | 2 | 0.009 |
| PAD | 5.87 | 1 | 0.015 |
| Type of Ulcer | 11.42 | 3 | 0.010 |

**Table S12**: **Univariate association of clinical factors with delayed healing** **(>5 months).** Delayed healing was defined as healing time >5 months among participants with available healing-time data (n=766). Pearson’s chi-square test was used for categorical variables.

| **Variable** | **Total N** | **Not delayed mean rank** | **Delayed mean rank** | **Mann–Whitney U** | **Wilcoxon W** | **p-value** |
| --- | --- | --- | --- | --- | --- | --- |
| Duration of ulcer | 671 | 313.45 | 379.52 | 40644 | 138547 | <0.001 |
| Duration of hospital stay | 764 | 339.59 | 465.68 | 43894.5 | 171154.5 | <0.001 |
| Number of reviews | 765 | 301.79 | 540.74 | 24637.5 | 152402.5 | <0.001 |

**Table S13: Independent-samples Mann–Whitney U test results comparing patients with and without delayed healing.** Delayed healing was defined as healing time >5 months.
